# NICE verdict on Temozolomide: where next?

**DOI:** 10.1038/sj.bjc.6600134

**Published:** 2002-02-12

**Authors:** M Brada

**Affiliations:** The Institute of Cancer Research and The Royal Marsden NHS Trust, Downs Road, Sutton, Surrey SM2 5PT, UK

## Abstract

*British Journal of Cancer* (2002) **86**, 499–500. DOI: 10.1038/sj/bjc/6600134 www.bjcancer.com

© 2002 Cancer Research UK

## 

Temozolomide is a UK product developed in a CRC laboratory at Aston University by Malcolm Stevens and his team. It came to clinical testing in the early 1990s and the first UK study suggested activity in recurrent malignant glioma beyond that perceived for conventional therapy (O'Reilly *et al*, 1993). This raised hope in a largely chemoresistant tumour with poor prognosis. However, the subsequent UK experience in a CRC multicentre phase II study in recurrent malignant glioma did not reproduce the results expected from the initial experience (Bower *et al*, 1997). Time moved on and Temozolomide was licenced to Schering Plough. Following three preregistration studies which were the largest ever performed in patients with recurrent malignant glioma (Yung *et al*, 1999, 2000; Brada *et al*, 2001) it became available in clinical practice. The median progression free survival and survival were 5.4 and 13.6 months in anaplastic astrocytoma and 3 and 7.3 months in glioblastoma with 35% response rate in anaplastic astrocytoma and 6–8% in glioblastoma multiforme and quality of life benefit. Two studies had no comparative group and one randomized Phase II study demonstrated small progression free survival gain compared to single agent Procarbazine with no survival benefit (Yung *et al*, 2000).

The introduction of a new chemotherapeutic agent in EU countries is through product authorization by the European Agency for the Evaluation of Medicinal Products (EMEA) which allows for sales for a specified indication. This however does not lead to free availability within the National Health Service. Currently the release of funding for new treatment is strongly influenced by a NICE (National Institute of Clinical Excellence) review.

It is reasonable to ask whether the NICE process is necessary following a full review by EMEA prior to drug registration. The answer is complex and highlights the different requirements and responsibilities of each of the organisations. It also reflects the different approaches to the introduction of new therapies in the UK and other EU member states.

Be that as it may, we are presented with a review commissioned by the Health Technology Assessment (HTA) programme on behalf of NICE and the summary is reported in this issue of the journal (Dinnes *et al*, this issue). From the publication it is clear that the structured evaluation of Temozolomide studies looked at issues of efficacy and toxicity in a different manner than EMEA. An authority granting a product licence asks whether an agent works and whether it is safe. The requirement is for ‘significant therapeutic innovation’ although the term is not fully defined. The remit of NICE evaluation is to provide guidance on clinical and cost effectiveness of new treatment. The attempt is to stay clear of political and commercial pressures and come up with as objective an assessment as is possible with vested interests hovering in the wings.

The systematic Cochrane type review of published articles (described in detail in the full publication (Dinnes *et al*, 2001) concludes that the evidence base for the use of Temozolomide in malignant glioma is ‘weak and few strong conclusions can be drawn regarding its effectiveness’ (Dinnes *et al*, this issue). This is in stark contrast to the description of the agent during its launch as ‘the most important advance in brain cancer treatment for the past 20 years’.

There is little disagreement that the last 20 years have not been a very productive period for the development of new treatments in malignant gliomas and it may well be that a new drug with promise is an addition which is sorely needed.

Temozolomide is an interesting agent with little toxicity and easy method of administration, but of limited efficacy in recurrent malignant gliomas, particularly glioblastoma multiforme. This is not surprising in the face of a tumour which has frustrated most therapeutic endeavours. In retrospect it is too much to expect for a new single agent, whose primary mode of action is a common alkylating path, to be highly effective in a largely chemoresistant tumour. Nevertheless selected patients with recurrent anaplastic astrocytoma with good performance status have an objective response rate higher than previously perceived for malignant glioma (Yung *et al*, 1999) although comparative information on such a selected cohort is not available.

While commercial interest is understandably directed to exploring the use of Temozolomide in common tumours we must not lose sight of the opportunity to define its role in the management of glial tumours. An important step missing from initial glioma studies is a head to head comparison with conventional treatment particularly at the time of recurrence. To this end a randomized CRC/NCRI study comparing Temozolomide and nitrosoureas is due to commence in early 2002. The EORTC brain tumour group and RTOG have also commenced randomized studies of single agent Temozolomide in primary malignant glioma concomitant with and adjuvant to radiotherapy.

There is also a need to determine the optimum scheduling of Temozolomide. A number of alternative schedules of administration have been investigated aiming for higher dose intensity and saturating DNA repair enzyme through prolonged and/or multiple day administration (Brock *et al*, 1998; Middleton *et al*, 2000; Spiro *et al*, 2001) but higher efficacy in clinical setting has not yet been demonstrated. Temozolomide is also under test in combination with a variety of other agents but the present design of phase II studies in malignant glioma has so far not come up with a clear winner (Gander *et al*, 1999; Britten *et al*, 1999; Patel *et al*, 2000). The potentially most promising avenue for increasing the efficacy of Temozolomide is through modulation of drug resistance. The two main resistance mechanisms for Temozolomide induced DNA damage are via demethylation through O^6^-alkylguanine DNA alkyltransferase (AGT) and repair by DNA mismatch repair protein (Baer *et al*, 1993; Liu *et al*, 1996; Wedge and Newlands, 1996). *In vitro* and *in vivo* depletion of AGT increases Temozolomide cytotoxicity (Wedge *et al*, 1997; Middleton *et al*, 1998, 2000). However published clinical studies showing convincing improvement in therapeutic ratio are not yet available.

The availability of Temozolomide has generated new research activity in a relatively unexplored tumour. Activity does not necessarily equate with progress and to accomplish real improvement we need to take a lesson from the past. Clinical trials conducted over the last 20 years aiming to evaluate new agents in the management of patients with malignant glioma have largely failed to find new effective treatment strategies. While this may be due to primary resistance of malignant glioma to therapy, it may also in part be due to inadequate trial design and inappropriate or difficult endpoints (Brada *et al*, 1999).

To some the assessment of Temozolomide may seem unduly harsh. Nevertheless it provides a clear message that the new agent is not a panacea. We should therefore avoid the trap of widespread use and concentrate on studies to optimize the potential of what is an interesting new oral chemotherapeutic agent. To grasp the opportunity to develop more effective therapy in malignant glioma incorporating Temozolomide will require robust and reliable study design coupled with intellectually honest interpretation of results. Quick fix is unlikely to avoid the failure of the last 20 years.
